# SARS-CoV-2 triggers inflammatory responses and cell death through caspase-8 activation

**DOI:** 10.1038/s41392-020-00334-0

**Published:** 2020-10-09

**Authors:** Shufen Li, Yulan Zhang, Zhenqiong Guan, Huiling Li, Meidi Ye, Xi Chen, Jun Shen, Yiwu Zhou, Zheng-Li Shi, Peng Zhou, Ke Peng

**Affiliations:** 1 State Key Laboratory of Virology, CAS Key Laboratory of Special Pathogens, Center for Biosafety Mega-Science, Wuhan Institute of Virology, Chinese Academy of Sciences, Wuhan, 430071 China; 2 University of Chinese Academy of Sciences, Beijing, China; 3 Department of Thoracic and Vascular Surgery, Wuhan No. 1 Hospital, Tongji Medical College, Huazhong University of Science and Technology, Wuhan, 430022 China; 4 Infectious Disease Department, Children’s Hospital of Fudan University, National Children’s Medical Center, Shanghai, 201102 China; 5 Department of Forensic Medicine, Tongji Medical College of Huazhong University of Science and Technology, Hangkong Road, Wuhan, 430074 China

**Keywords:** Infectious diseases, Inflammation

## Abstract

Severe acute respiratory syndrome coronavirus 2 (SARS-CoV-2) infection can lead to respiratory illness and multi-organ failure in critically ill patients. Although the virus-induced lung damage and inflammatory cytokine storm are believed to be directly associated with coronavirus disease 2019 (COVID-19) clinical manifestations, the underlying mechanisms of virus-triggered inflammatory responses are currently unknown. Here we report that SARS-CoV-2 infection activates caspase-8 to trigger cell apoptosis and inflammatory cytokine processing in the lung epithelial cells. The processed inflammatory cytokines are released through the virus-induced necroptosis pathway. Virus-induced apoptosis, necroptosis, and inflammation activation were also observed in the lung sections of SARS-CoV-2-infected HFH4-hACE2 transgenic mouse model, a valid model for studying SARS-CoV-2 pathogenesis. Furthermore, analysis of the postmortem lung sections of fatal COVID-19 patients revealed not only apoptosis and necroptosis but also massive inflammatory cell infiltration, necrotic cell debris, and pulmonary interstitial fibrosis, typical of immune pathogenesis in the lung. The SARS-CoV-2 infection triggered a dual mode of cell death pathways and caspase-8-dependent inflammatory responses may lead to the lung damage in the COVID-19 patients. These discoveries might assist the development of therapeutic strategies to treat COVID-19.

## Introduction

Since its emergence in December 2019, the severe acute respiratory syndrome coronavirus 2 (SARS-CoV-2) has caused a pandemic transmission in the world. Virus infection causes severe respiratory illness in the patients, called the coronavirus disease 2019 (COVID-19).^1^ As of 24 July 2020, SARS-CoV-2 had infected over 15 million people worldwide and had claimed the death of over 630,000 people. Severe disease manifested by fever and pneumonia, leading to acute respiratory distress syndrome (ARDS), has been described in up to 20% of COVID-19 cases.^2^ Elevated levels of serum interleukin (IL)-6 and other inflammatory cytokines correlate with respiratory failure, ARDS, and adverse clinical outcomes.^3,4^ Inflammatory responses from infected cells may further induce infiltration of immune cells into the lung causing overproduction of pro-inflammatory cytokines resulting in severe lung damage and multi-organ dysfunction.^5^ Indeed, virus-induced cytokine storm and sepsis has claimed the death of 28% of fatal COVID-19 cases.^6^ Understanding the underlying molecular mechanisms of virus-induced inflammatory responses would be highly valuable for developing effective therapeutic strategies for treating COVID-19 patients.

Caspase-8, which was previously viewed exclusively as an apoptotic caspase, has now emerged as a master regulator of the three major cell death pathways, including apoptosis, pyroptosis, and necroptosis.^7^ Recently, it was reported that caspase-8 can induce the expression of pro-inflammatory cytokines and process pro-IL-1β and IL-18 in the same way as caspase-1, resulting in the release of bioactive cytokines, through either pyroptosis or necroptosis.^8^ The role of caspase-8 in mediating inflammatory responses has been reported in the context of infection with fungal pathogens such as *Candida albicans* and *Aspergillus fumigatus*.^9^ Whether caspase-8 activation can also mediate virus-induced inflammatory responses has not been reported before.

Necroptosis is an immunogenic cell death pathway that can eliminate virus-infected cells and mobilize both innate and adaptive immune responses to restrict virus replication.^10^ The benefits of necroptosis to the host, however, may sometimes be outweighed by the potentially deleterious hyper-inflammatory consequences of activating this death pathway in pulmonary and other tissues.^11^ For example, the influenza A virus (IAV) infection-induced necroptosis in airway epithelial cells is associated with lung damage and hyper-inflammatory responses. Similar with IAV, SARS-CoV-2 can cause severe lung damage and disease-associated hyper-inflammatory responses.^12^ Whether SARS-CoV-2 infection also induces necroptosis pathway to trigger cell death and inflammatory responses are currently unknown.

Here we report that SARS-CoV-2 infection of lung epithelial cells induces caspase-8 activation that triggers cell apoptosis and processing of inflammatory cytokines including IL-1β into the bioactive form. IL-1β was then secreted through the SARS-CoV-2-induced necroptosis pathway resulting in inflammatory responses. The SARS-CoV-2 infection induced a dual mode of cell death pathways, apoptosis, and necroptosis, and inflammatory responses were also observed in the infected HFH4-hACE2 transgenic mouse model and in the postmortem lung sections of fatal COVID-19 patients. Thus, the cell death and inflammatory responses are intimately linked during SARS-CoV-2 infection. The inflammatory responses from the infected epithelial cells may further induce infiltration of inflammatory cells inducing strong immune pathogenesis as revealed in the postmortem lung sections of fatal COVID-19 patients. These discoveries have shed light on the underlying mechanisms of COVID-19 pathogenesis and will offer valuable insights for the rational development of effective therapeutic strategies to treat COVID-19.

## Results

### SARS-CoV-2 infection induces inflammatory responses in lung epithelial cells

**Fig. 1:**
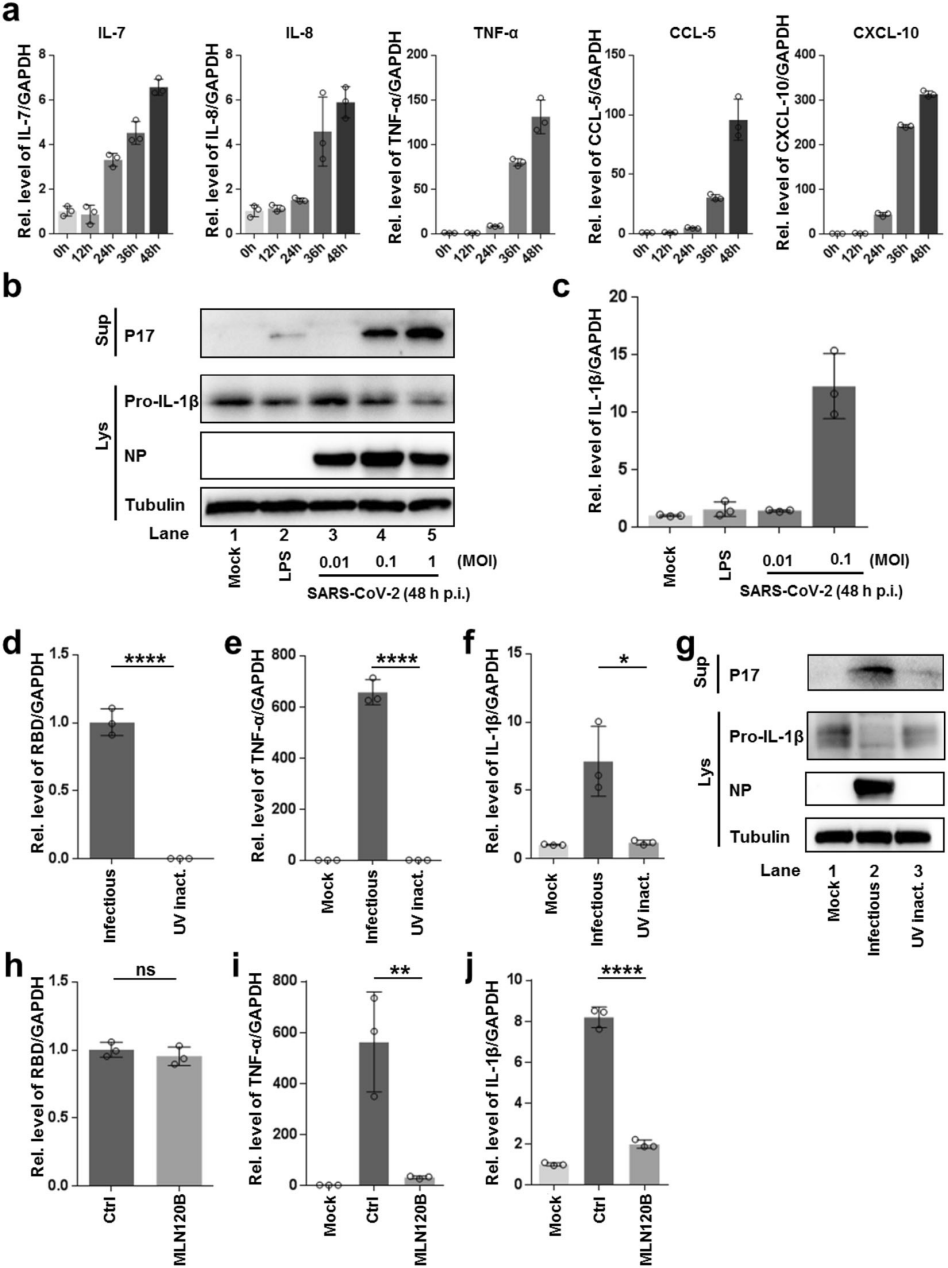
SARS-CoV-2 infection induces inflammatory responses through activation of the NFκB pathway. **a** Calu-3 cells were infected with SARS-CoV-2 (MOI = 0.1) with indicated time; intracellular mRNA levels of IL-7, IL-8, TNF-α, CCL5, and CXCL10 were measured with quantitative RT-PCR (qRT-PCR). **b**, **c** Calu-3 cells were treated with LPS (5 μg/ml) or infected with SARS-CoV-2 with indicated MOIs for 48 h. Mature IL-1β (P17) levels in the supernatants and intracellular levels of pro-IL-1β were determined by western blotting (**b**). Intracellular mRNA levels of IL-1β were measured with qRT-PCR (**c**). **d**–**g** Calu-3 cells were infected with SARS-CoV-2 (MOI = 0.1) or inoculated with UV-inactivated virus (equal amount) for 48 h. Intracellular mRNA levels of viral RBD (**d**),TNF-α (**e**), and IL-1β (**f**) were determined with qRT-PCR. P17 levels in the supernatants and intracellular levels of pro-IL-1β were determined by western blotting (**g**). **h**–**j** Calu-3 cells were treated with MLN120B (10 μM) followed by SARS-CoV-2 (MOI = 0.1) infection for 48 h. Intracellular mRNA levels of RBD (**h**), TNF-α (**i**), and IL-1β (**j**) were determined with qRT-PCR. Experiments were performed in triplicates. Data shown are means ± SD. Comparison of mean values (**d**–**f**, **h**–**j**) between two groups was analyzed by Student’s *t*-test. *p < 0.05; **p < 0.01; ****p < 0.0001; NS, no significance

To investigate the mechanisms of SARS-CoV-2-induced inflammatory responses, Calu-3 cells, a lung epithelial cell model, were infected and the cell lysates were collected at different time points post infection. Quantitative reverse-transcriptase PCR (qRT-PCR) analysis showed a time-dependent upregulation of inflammatory cytokines and chemokines including IL-7, IL-8, tumor necrosis factor-α (TNF-α), CXCL10, and CCL5 in the virus-infected cells (Fig. 1a), of which upregulation were also reported in the COVID-19 patients.^13^ Furthermore, SARS-CoV-2 infection of Calu-3 cells induced IL-1β processing and secretion in an multiplicity of infection (MOI)-dependent manner (Fig. 1b). The expression level of pro-IL-1β was also upregulated in the infected cells (Fig. 1c). To analyze whether SARS-CoV-2-induced inflammatory responses depend on viral replication, SARS-CoV-2 was inactivated with ultraviolet (UV) treatment. UV inactivation abolished virus replication (Fig. 1d), virus-induced upregulation of TNF-α and IL-1β (Fig. 1e, f), and also prevented pro-IL-1β processing and secretion (Fig. 1g). On the other hand, treatment with MLN120B, an inhibitor of nuclear factor-κB (NFκB), inhibited virus-induced upregulation of pro-IL-1β and TNF-α without affecting viral replication (Fig. 1h–j). Taken together, these results suggested that SARS-CoV-2-induced inflammatory responses depend on viral replication and activation of the NFκB pathway.

### SARS-CoV-2-induced IL-1β maturation and secretion depends on caspase-8 activation

**Fig. 2:**
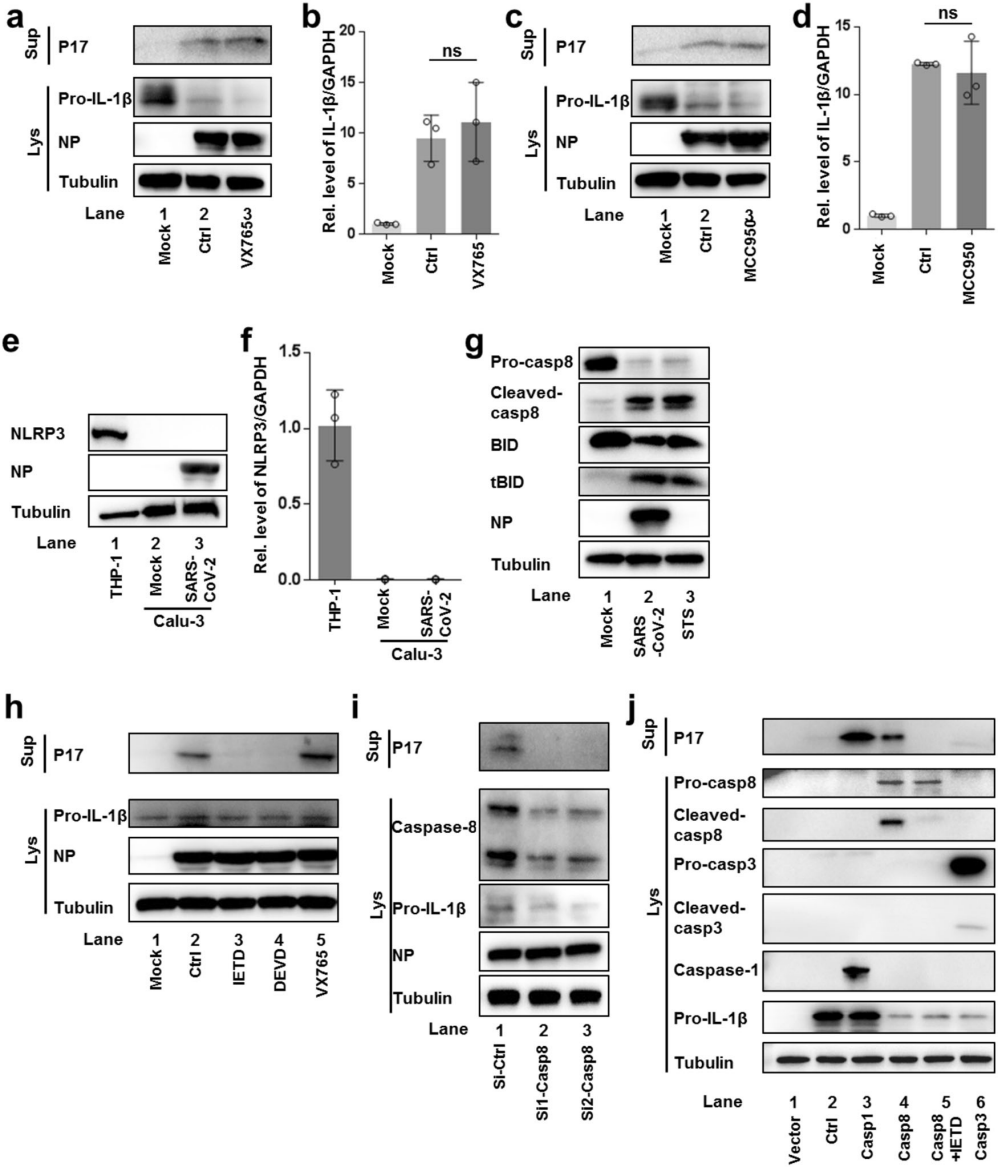
SARS-CoV-2 infection induces caspase-8 activation to mediate pro-IL-1β processing. **a**, **b** Calu-3 cells pretreated with VX-765 (20 μM) were infected with SARS-CoV-2 (MOI = 0.1) for 48 h. P17 levels in the supernatants and intracellular levels of pro-IL-1β were determined by western blotting (**a**). Intracellular mRNA levels of IL-1β were measured by qRT-PCR (**b**). **c**, **d** Calu-3 cells pretreated with MCC950 (100 μM) were infected with SARS-CoV-2 (MOI = 0.1) for 48 h. P17 levels in the supernatants and intracellular levels of pro-IL-1β were determined by western blotting (**c**). Intracellular mRNA levels of IL-1β were measured by qRT-PCR (**d**). **e**, **f** Calu-3 cells were mock-treated or infected with SARS-CoV-2 (MOI = 0.1); NLRP3 levels were determined by western blotting (**e**) or qRT-PCR (**f**) with THP-1 cells as a control. **g** Calu-3 cells infected with SARS-CoV-2 (MOI = 0.1) for 48 h were subjected to western blotting using the indicated antibodies. Cells treated with staurosporine (STS, 1 μM) for 24 h were used as a positive control. **h** Calu-3 cells were treated with Z-IETD-FMK (IETD, 50 μM), Z-DEVD-FMK (DEVD, 50 μM), or VX-765 (50 μM) followed by SARS-CoV-2 (MOI = 0.03) infection. P17 levels in the supernatants and intracellular levels of pro-IL-1β were determined by western blotting. **i** Calu-3 cells were transfected with siRNAs against caspase-8 for 48 h followed by SARS-CoV-2 (MOI = 0.1) infection. Cells and supernatants were collected at 48 h post infection. P17 levels in the supernatants and intracellular levels of pro-IL-1β were determined by western blotting. **j** Vero cells were co-transfected with plasmid expressing caspase-1, caspase-8, or caspase-3 and plasmid expressing IL-1β. Transfected cells were treated with Z-IETD-FMK (IETD, 50 μM) or left untreated. Twenty-four hours post transfection, the supernatants and cells were collected for western blotting analysis. Experiments were performed in triplicates. Data shown are means ± SD. Comparison of mean values (**b**, **d**) between two groups were analyzed by Student’s *t*-test. NS, no significance

We next analyzed the mechanism that mediate IL-1β processing and secretion. Activation of caspase-1 through the NLRP3 inflammasome is often involved in virus-induced IL-1β processing and secretion.^14^ However, treatment with the caspase-1 inhibitor, VX-765, did not affect SARS-CoV-2-induced pro-IL-1β production or IL-1β processing and secretion (Fig. 2a, b). Similar results were observed with treatment of the NLRP3 inhibitor MCC950 (Fig. 2c, d). Furthermore, both western blotting and quantitative PCR (qPCR) assays showed that NLRP3 is undetectable in the Calu-3 cells (Fig. 2e, f). Together, these results indicated that SARS-CoV-2-induced IL-1β processing and secretion is independent of caspase-1 and NLRP3 inflammasome in the Calu-3 cells.

It was recently reported that caspase-8 can mediate IL-1β maturation through direct cleavage of the pro-IL-1β at the same site with caspase-1.^15^ We found that SARS-CoV-2 infection of Calu-3 cells induced pronounced caspase-8 cleavage (Fig. 2g). The cleavage of BH3-interacting domain death agonist (BID), a substrate of caspase-8, into the truncated form further confirmed activation of caspase-8 in the SARS-CoV-2-infected cells (Fig. 2g). To analyze whether activated caspase-8 mediated SARS-CoV-2-induced inflammatory responses, Calu-3 cells were infected by SARS-CoV-2 in the presence of inhibitors against different caspases. The inhibition effects of these inhibitors were verified (Supplementary Fig. S1). The treatment of caspase-8-specific inhibitor Z-IETD-FMK strongly reduced IL-1β processing and secretion in the SARS-CoV-2-infected cells without affecting viral replication (Fig. 2h). Similar results were observed for Z-DEVD-FMK treatment, which has a broader inhibition effects against caspase-3, caspase-6, caspase-7, caspase-8, and caspase-10 (Fig. 2h). Consistent with the caspase-8 inhibitor treatment, depletion of caspase-8 with small interfering RNA (siRNA) also reduced the processing and secretion of IL-1β without affecting virus replication (Fig. 2i).

We further analyzed processing of pro-IL-1β through co-transfection experiments. As shown in Fig. 2j, co-transfection of pro-IL-1β with caspase-1 but not caspase-3 resulted in IL-1β processing and secretion. Co-transfection of caspase-8 with pro-IL-1β resulted in IL-1β processing and secretion similarly with caspase-1, which was inhibited by treatment with caspase-8 inhibitor Z-IETD-FMK as previously reported^15^ (Fig. 2j). These results suggested that SARS-CoV-2 infection induces caspase-8 activation to mediate pro-IL-1β processing.

### SARS-CoV-2-induced IL-1β secretion depends on the necroptosis pathway

**Fig. 3:**
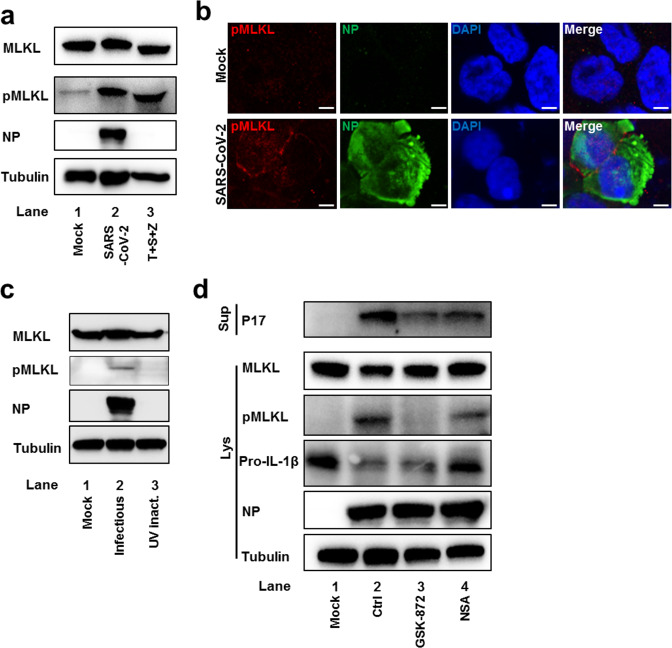
SARS-CoV-2 activates the necroptosis pathway to mediate IL-1β secretion. **a** Calu-3 cells were mock-treated or infected with SARS-CoV-2 (MOI = 0.1) for 48 h and subjected to western blotting using the indicated antibodies. Cells treated with 100 ng/ml of TNF-α, 50 nM of SM-164, and 100 μM of Z-VAD-FMK (T + S + Z) for 48 h were used as a positive control. **b** Mock-treated or SARS-CoV-2 (MOI = 0.1)-infected Calu-3 cells were fixed at 36 h post infection, followed by staining with antibodies against pMLKL (red) and viral antigen NP (green). Nuclei were stained by DAPI (blue). Scale bar: 5 μm. **c** Calu-3 cells were infected with SARS-CoV-2 (MOI = 0.1) or inoculated with UV-inactivated virus (equal amount) for 48 h and subjected to western blotting using the indicated antibodies. **d** Calu-3 cells pretreated with GSK-872 (5 μM) or NSA (2.5 μM) were infected with SARS-CoV-2 (MOI = 0.1) for 48 h. P17 levels in the supernatants and intracellular levels of MLKL, pMLKL, and pro-IL-1β were determined by western blotting

We further analyzed the pathway that mediates IL-1β secretion during SARS-CoV-2 infection. The independence of caspase-1 activity for SARS-CoV-2-induced IL-1β processing and secretion suggests that pyroptosis might not play a major role during IL-1β secretion. Necroptosis is another form of cell death that can mediate secretion of inflammatory cytokines including IL-1β.^16^ We therefore analyzed whether SARS-CoV-2 can trigger necroptosis in the Calu-3 cells. The mixed lineage kinase domain-like (MLKL) is the effector of necroptosis and is phosphorylated by the receptor-interacting protein kinase-3 (RIPK3) during necroptosis. SARS-CoV-2 infection of Calu-3 cells induced phosphorylation of MLKL (pMLKL) similar with the positive control (Fig. 3a). Staining with the antibody against pMLKL confirmed the upregulation of pMLKL in the infected cells and showed a staining pattern on the plasma membrane, consistent with the localization of activated pMLKL (Fig. 3b). UV inactivation of SARS-CoV-2 prevented virus replication and virus-induced MLKL phosphorylation, indicating of viral replication-dependent activation of the necroptosis pathway (Fig. 3c). Inhibition with RIPK3 inhibitor GSK-872 inhibited pMLKL in the infected cells, suggesting that SARS-CoV-2 induces RIPK3 phosphorylation to trigger the necroptosis pathway (Fig. 3d). Moreover, treatment with RIPK3 inhibitor GSK-872 reduced IL-1β maturation and secretion without affecting viral replication (Fig. 3d and Supplementary Fig. S2). Similarly, treatment with necrosulfonamide (NSA), a pMLKL membrane translocation inhibitor, reduced IL-1β maturation and secretion without affecting viral replication, confirming the role of necroptosis in SARS-CoV-2-induced inflammatory responses (Fig. 3d).

### SARS-CoV-2 infection triggers apoptosis through caspase-8 activation

**Fig. 4:**
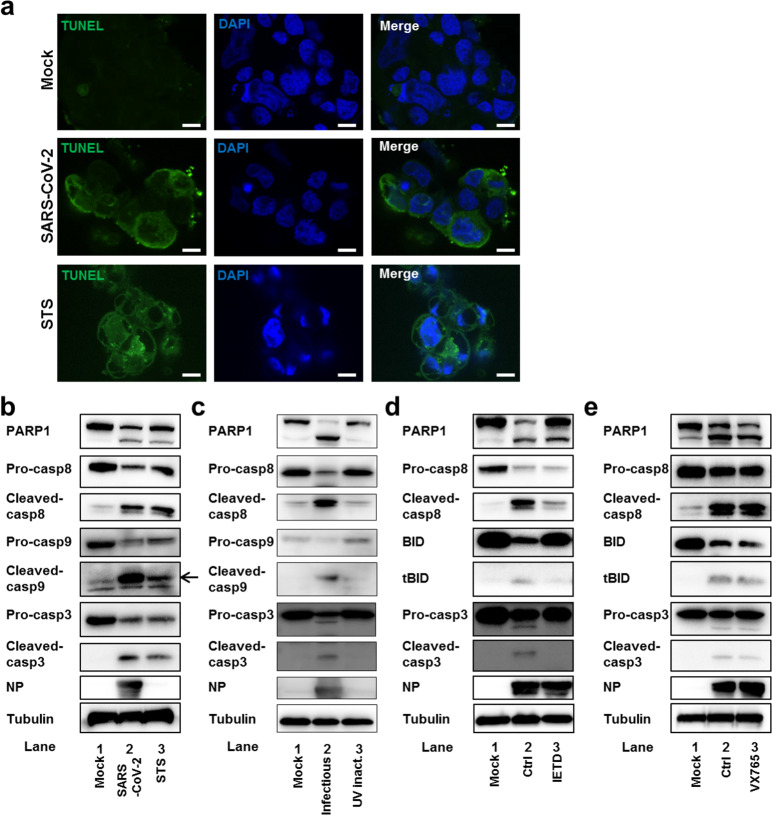
SARS-CoV-2 infection triggers apoptosis through caspase-8 activation. **a** Mock-treated, SARS-CoV-2 (MOI = 0.1)-infected, or STS (1 μM)-treated Calu-3 cells were fixed at 36 h post infection, followed by labeling with TUNEL (green) and staining with DAPI (blue). Scale bar: 10 μm. **b** Mock-treated or SARS-CoV-2 (MOI = 0.1)-infected Calu-3 cells were collected at 48 h post infection and subjected to western blotting using the indicated antibodies. Cells treated with staurosporine (STS, 1 μM) for 24 h were used as a positive control. The arrow indicates the specific band of cleaved caspase-9. **c** Calu-3 cells infected with SARS-CoV-2 (MOI = 0.1) or inoculated with UV-inactivated virus (equal amount) were collected at 48 h post infection and subjected to western blotting using the indicated antibodies. **d** Calu-3 cells pretreated with Z-IETD-FMK (IETD, 50 μM) were infected with SARS-CoV-2 (MOI = 0.1) for 48 h and subjected to western blotting using the indicated antibodies. **e** Calu-3 cells pretreated with VX-765 (50 μM) were infected with SARS-CoV-2 (MOI = 0.1) for 48 h and subjected to western blotting using the indicated antibodies

Caspase-8 is known to trigger apoptosis through both the extrinsic and intrinsic pathways.^17^ To assess whether SARS-CoV-2 infection induced cell apoptosis in lung epithelial cells, the terminal deoxynucleotidyl transferase dUTP nick end labeling (TUNEL) assay was performed on SARS-CoV-2-infected Calu-3 cells. TUNEL staining revealed an increased number of apoptotic cells in infected cells, compared with the mock control cells, indicating activation of cellular apoptosis (Fig. 4a). Western blotting analysis showed that caspase-8, caspase-9, and caspase-3 were cleaved into the activated forms in SARS-CoV-2-infected cells, similarly as in the control cells treated with staurosporine (Fig. 4b). In addition, cleavage of the poly (ADP-ribose) polymerase 1 (PARP-1), a hallmark of apoptosis activation, was observed in the infected cells (Fig. 4b). In cells that were infected by the UV-inactivated SARS-CoV-2 virus, the cleavage of caspase-8, caspase-9, caspase-3, and PARP-1 was prevented, indicating that the apoptosis was dependent on viral replication (Fig. 4c). Treatment with caspase-8-specific inhibitor Z-IETD-FMK inhibited virus-induced BID cleavage, caspase-3 activation, and PARP-1 cleavage (Fig. 4d). In contrast, treatment with caspase-1 inhibitor, VX-765, did not show such an inhibition (Fig. 4e). These results suggested that SARS-CoV-2-triggered apoptosis depends on viral replication and caspase-8 activation.

### SARS-CoV-2 infection induces pronounced cell death and inflammatory responses in the HFH4-hACE2 mouse model and human postmortem lung section

**Fig. 5:**
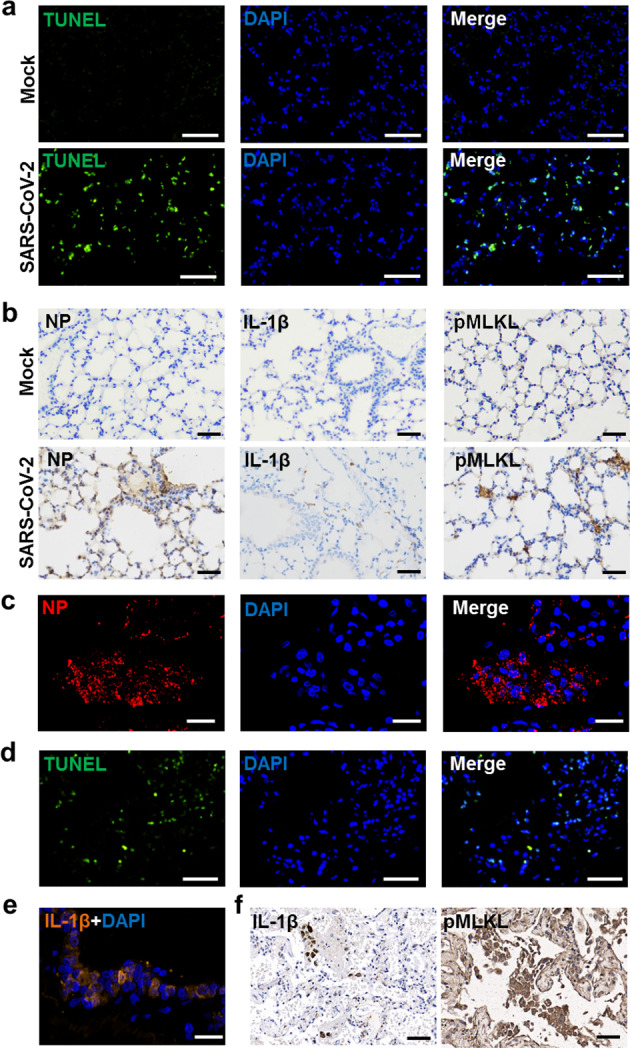
SARS-CoV-2 induces cell death and inflammatory responses in the HFH4-hACE2 mouse model and human postmortem lung. **a**, **b** Paraffin-embedded lung sections from mock-treated or SARS-CoV-2-infected HFH4-hACE2 mice (3 × 10^4^ TCID_50_) at 3 days post infection were labeled with TUNEL (green) and stained with DAPI (blue) (**a**), or subjected to immunohistochemistry with the indicated antibodies (**b**). **c**–**f** Human postmortem lung sections were prepared from a fatal COVID-19 patient. Sections were stained with viral antigen SARS-CoV-2-NP (Red) and DAPI (blue) (**c**), or labeled with TUNEL (green) and stained with DAPI (blue) (**d**), or stained with IL-1β (orange) and DAPI (blue) (**e**), or subjected to immunohistochemistry with indicated antibodies (**f**). **a**, **b**, **d**, **f** Scale bar: 50 μm. **c**, **e** Scale bar: 20 μm

We further analyzed whether SARS-CoV-2 infection induces inflammatory responses and cell death in the HFH4-hACE2 mouse model, a recently established animal model for studying the pathogenesis of SARS-CoV-2.^18^ The HFH4-hACE2 mice were intranasally infected with SARS-CoV-2 or mock-treated and the lung tissue was collected at 3 days post infection. TUNEL staining revealed a large number of apoptotic cells in the lung section of the infected mice but not in that of the mock-infected mice, indicating that SARS-CoV-2 infection induces pronounced apoptosis in the lung of infected mice (Fig. 5a). Immunohistochemical assays showed strong staining of virus NP protein in the lung section, confirming the productive viral infection of the lung tissues (Fig. 5b). IL-1β was also detected in the lung section of the infected mice but not in that of the mock control, confirming virus-induced inflammatory responses (Fig. 5b). Importantly, staining with the antibody against pMLKL revealed strong signals in the lung section of infected mouse but not in that of the mock control (Fig. 5b). These results suggested that SARS-CoV-2 infection of the HFH4-hACE2 mouse model leads to pronounced apoptosis and necroptosis concomitantly with inflammatory responses in the lung.

**Fig. 6:**
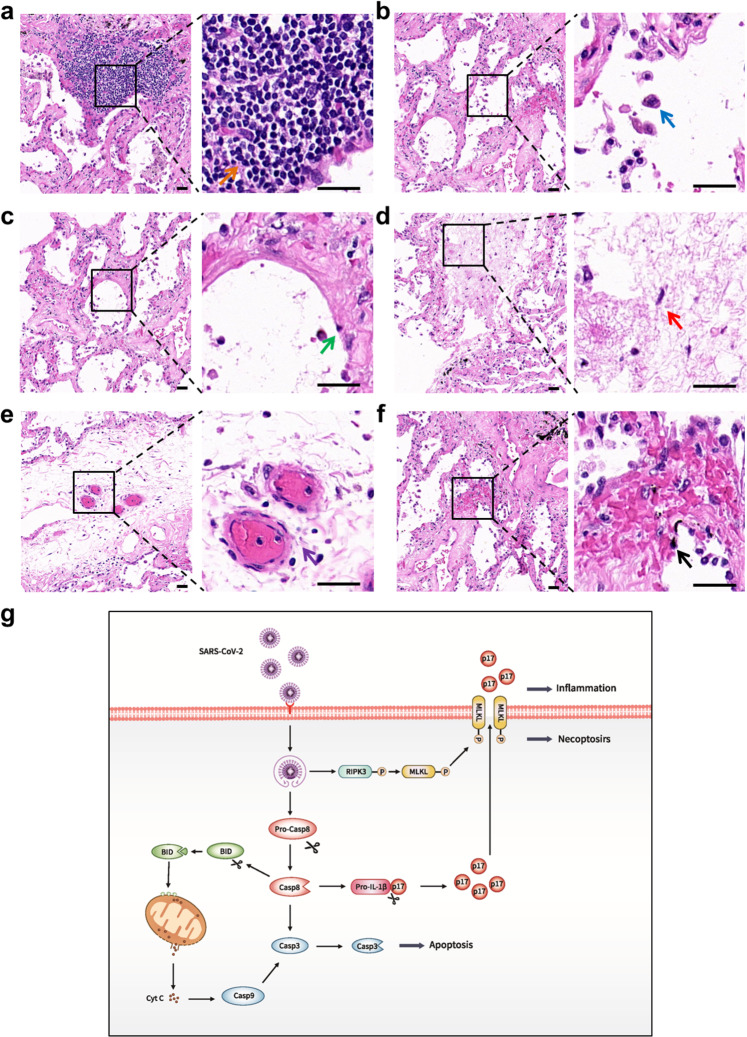
Pathological changes in human postmortem lung and a proposed model of SARS-CoV-2 infection induces inflammatory responses and cell death. **a**–**f** Sections prepared from human postmortem lung were stained with hematoxylin and eosin to observe pathological changes. Lymphocyte aggregation (**a**, yellow arrow), macrophage infiltration (**b**, blue arrow), necrotic cell debris (**c**, green arrow), early fibrosis formation (**d**, red arrow), extravasated blood (**e**, purple arrow), and hemorrhage (**f**, black arrow) were observed in the lung sections. Right panels are enlarged pictures of the boxed regions. Scale bar, 25 μm. **g** A proposed model of SARS-CoV-2 infection induces inflammatory responses and cell death. SARS-CoV-2 infection induces the cell death through the activation of caspase-8. The activation of caspase-8 promotes pro-IL-1β cleavage, leading to the secretion of mature IL-1β (P17) through necroptosis pathway

Finally, we analyzed the SARS-CoV-2 infection in the postmortem lung sections from fatal COVID-19 patients. Staining with the antibody against virus NP protein confirmed viral infection in the lung section (Fig. 5c). TUNEL assay performed on the lung section revealed distinct signals, indicating pronounced cell apoptosis similar with the situation in the infected HFH4-hACE2 mouse model (Fig. 5d). Staining with antibodies against IL-1β and pMLKL revealed that strong inflammation and necroptosis were induced in the lung tissue (Fig. 5e, f). Consistent with the virus-induced immunopathogenesis, severe interstitial inflammatory cell aggregation and infiltration (Fig. 6a, b), necrotic cell debris (Fig. 6c), early fibrosis formation (Fig. 6d), extravasated blood (Fig. 6e), and hemorrhage (Fig. 6f) were observed in the postmortem lung sections from fatal COVID-19 patients through hematoxylin-eosin (HE) staining assay. Taken together, these results suggested that SARS-CoV-2 infection induces cell death concomitantly with inflammatory responses and necroptosis activation in the lung tissue of the COVID-19 patients.

## Discussion

Here we report that SARS-CoV-2 infection of lung epithelial cells triggers cell death and inflammatory responses through the activation of caspase-8. Caspase-8 is a master regulator of several cell death pathways, including apoptosis, necroptosis, and pyroptosis.^19^ Its role in regulation of inflammatory responses has been recently reported in the context of fungal infection. We found that SARS-CoV-2 induces caspase-8 activation to trigger cell apoptosis and to directly process inflammatory factors such as pro-IL-1β. The processed IL-1β is then secreted through the SARS-CoV-2-triggered necroptosis pathway (Fig. 6g). Thus, these discoveries have expanded the role of caspase-8 in the regulation of inflammatory responses to the context of virus infection.

The caspase-8-mediated apoptosis activation and inflammatory responses in infected lung epithelial cells may induce downstream immune pathogenesis in the lung tissue. In line with this notion, massive infiltration of inflammatory cells, necrotic cell debris, and pulmonary interstitial fibrosis were observed in the postmortem lung sections of fatal COVID-19 patients. TUNEL and immunohistochemistry (IHC) staining revealed pronounced apoptosis and necroptosis in the lung sections. Together, these mechanisms might lead to severe lung damage and immune pathogenesis during SARS-CoV-2 infection. This suggests that caspase-8 activation plays a central role in SARS-CoV-2-induced apoptosis and inflammatory responses. As the other two highly pathogenic coronaviruses, the SARS-CoV and MERS-CoV, also infect the lower respiratory track and cause severe pneumonia in patients; whether similar mechanisms were conserved among these viruses may be interesting questions for further investigation.

Necroptosis is a form of immunogenic cell death that can trigger inflammatory responses through releasing inflammatory cytokines and cellular damage associated molecular patterns (DAMPs).^20^ Although SARS-CoV-2 activates caspase-8 to process pro-IL-1β, high level of caspase-8 activation is known to inhibit necroptosis pathway mediated by the RIPK3 and MLKL.^21^ Thus, although caspase-8 activation is important for processing of pro-IL-1β in the context of SARS-CoV-2 infection, its activation did not reach a level that fully inhibited necroptosis and prevented IL-1β secretion. This suggests that SARS-CoV-2 induced a regulated level of caspase-8 activation to process pro-IL-1β and, meanwhile, to allow sufficient necroptosis activation for IL-1β release. Further efforts are needed to understand the mechanisms of how does SARS-CoV-2 trigger the necroptosis pathway and achieve the regulated caspase-8 activation to induce the inflammatory responses. The SARS-CoV-2-induced inflammatory responses were reported to be a major determinant for the disease progression in COVID-19 patients.^22^ As blocking necroptosis strongly inhibited SARS-CoV-2-induced inflammatory responses, whether anti-necroptosis treatment can be integrated into the therapeutic strategies to treat COVID-19 warrants further evaluation. It should also be noted that the necroptosis inhibitor did not fully block IL-1β secretion during SARS-CoV-2 infection, indicating that other pathways, such as pyroptosis, may also be involved in the inflammatory responses. Future efforts can be devoted to investigate whether pyroptosis and activation of gasdermins also play a role during SARS-CoV-2 inflammation.

Depending on the status of activation, the virus-triggered cell death pathways might also be beneficial to the host. It has been reported that influenza virus infection triggers both apoptosis and necroptosis in the lung as redundant cell death processes resulting in immune responses to restrict viral replication.^11^ However, when these two pathways were triggered to a hyper-activation status, IAV infection leads to severe lung damage and fatal infection outcome.^11^ Similar with IAV, the SARS-CoV-2 induced a dual mode of cell death pathways in the lung epithelial cells, which may pose anti-viral responses or immune pathogenesis depending on the activation status. Many factors may contribute to the level of cell death activation, including initial dose of viral infection, the underlying co-morbidity in the patient, age factor, etc. It would be important to study the regulated cell death activation and other concurrent factors in the clinical settings that determine the infection outcome of COVID-19 patients.

## Materials and methods

### Specimen collection and ethics

Post mortem lung sections were prepared from a patient who died of COVID-19. The specimen collection and research was approved by the ethics committee of Wuhan Institute of Virology (WIV) and the designated hospital for COVID-19. The mice infection study was approved by WIV animal welfare committee.

### Cell lines

Calu-3 cells were obtained from American Type Culture Collection (ATCC) and cultured in minimum Eagle’s medium (MEM; Gibco) supplemented with 10% fetal bovine serum (FBS; Gibco), 1% MEM non-essential acids (Gibco), 1% sodium pyruvate (100 mM, Gibco), and 1% antibiotics (Gibco). Vero and Vero E6 cells were obtained from ATCC and maintained in Dulbecco’s modified Eagle’s medium (Gibco) supplemented with 10% FBS and 1% antibiotics. THP-1 cells obtained from ATCC were maintained in RPMI-1640 medium containing 10% FBS and antibiotics. Cells were cultured at 37 °C in a humidified atmosphere of 5% CO_2_.

### Viruses

The SARS-CoV-2 (IVCAS 6.7512) was isolated from the bronchoalveolar lavage fluid sample of patient^23^ and was propagated in Vero E6 cells. Viral titer (TCID_50_) was determined on Vero E6 cells. Briefly, confluent monolayers were incubated with tenfold serial dilutions of virus for 3 days. Then the cytopathic effect was observed and the TCID_50_ was calculated according to the Reed-Muench formula. Virus was inactivated by exposing to UV light for 1 h and the inactivation was confirmed by titer determination on Vero E6 cells.

### Antibodies and reagents

Monoclonal rabbit anti-NLRP3 (D2P5E, 13158 S), anti-caspase-9 (9502 T), anti-caspase-8 (1C12, 9746 S), anti-caspase-3 (9665 S), anti-cleaved-caspase-3 (Asp175, 9661S), anti-BID (2002S), anti-caspase-1 (2225S), anti-MLKL (14993S), anti-pMLKL (S358, used in western blotting, 91689S), and anti-PARP-1 (9542S) were purchased from Cell Signaling Technology (Beverly, MA, USA). Polyclonal antibody anti-IL-1β (A1112) was acquired from Abclonal (Wuhan, China). Polyclonal rabbit anti-α-tubulin (11224-1-AP) was purchased from Proteintech (Chicago, IL, USA). Anti-pMLKL (S358, used in immunofluorescence assay and IHC, ab187091) was purchased from Abcam (Cambridge, UK). Anti-pMLKL (S345, used in IHC, MA5-32752) was purchased from Thermo Fisher (Waltham, MA, USA). Rabbit anti-RP3-NP antibody (RP3) and mouse-anti-SARS-CoV-2-NP (SR24) were made in-house.

LPS (L4391) was obtained from Sigma-Aldrich (St. Louis, MO, USA). VX-765 (S222), MCC950 (S7809), Z-VAD-FMK (S7023), Z-IETD-FMK (S7314), Z-DEVD-FMK (S7312), and NSA (S8251) were obtained from Selleck (Houston, TX, USA). MLN120B (HY-15473), GSK-872 (HY-101872), SM-164 (HY-15989), and staurosporine (HY-15141) were purchased from MedChemExpress (New Jersey, USA). TNF-α (300-01A) was obtained from Peprotech (Rocky Hill, NJ, USA). DAPI (4,6-diamidino-2-phenylindole, C1002) was purchased from Beyotime (Shanghai, China).

### Plasmids construction

For the construction of plasmids expressing caspase-3 and caspase-8 (pRK-caspase-3-strep and pRK-casapse8-strep), cDNAs encoding human caspase-3 (NM_001354777.1) and caspase-8 (NM_033355.3) were synthesized by Tsingke (Wuhan, China) and cloned into pRK plasmid with a C-terminal strep tag. pcDNA3.1-FLAG-IL-1β and pcDNA3.1-FLAG-caspase-1 were kindly provided by Dr. Jun Cui (Sun Yat-sen University).

### siRNA transfection

Calu-3 cells seeded in 24-well plates (2 × 10^5^ cells per well) were transfected with 40 pmol of siRNAs using Lipofectamine™ RNAiMAX Transfection Reagent (Invitrogen) according to the manufacturer’s protocols with NC siRNA as a control. At 48 h post transfection, cells were infected with SARS-CoV-2 (MOI = 0.1) or were mock-infected. Cells and supernatants were collected for further analyses at 48 h post infection. The siRNA oligonucleotides were synthesized by GenePharma (Suzhou, China) with the sequences as follows:

si1-Casp8: 5′-ACAUGAACCUGCUGGAUAUTT-3′;

si2-Casp8: 5′-GGACUUCAGCAGAAAUCUUTT-3′;

si-NC: 5′-UUCUCCGAACGUGUCACGUTT-3′.

### RNA isolation and qRT-PCR analysis

Total RNAs in collected cells were extracted with RNAiso Plus (TAKARA, Japan) following the manufacturer’s instructions. qRT-PCR was performed with a two-step procedure using the HiScript III 1st Strand cDNA Synthesis Kit (Vazyme, China) and ChamQ Universal SYBR qPCR Master Mix (Vazyme, China). Primer sequences are available upon request.

### Western blotting analysis

Cells treated and collected as indicated were lysed with cell lysis buffer (Beyotime, China). Cell lysates were subjected to 10–15% sodium dodecyl sulfate (SDS)-polyacrylamide gel electrophoresis and further transferred to polyvinylidene difluoride membranes (Millipore). After blocking, proteins were blotted with the indicated primary antibodies and then corresponding horseradish peroxidase-conjugated secondary antibodies. Protein bands were detected by an enhanced chemiluminescence kit (Millipore) using a Chemiluminescence Analyzer (Chemiscope600pro).

### Mature IL-1β detection

Proteins in 500 μl of the collected supernatant were precipitate with equal volume of methanol and a quarter volume of chloroform. The precipitant was dissolved in 2 × SDS loading buffer for western blotting analyses as described previously using antibodies against IL-1β (ABclonal, China).

### Co-transfection assay

Vero cells pre-seeded in 12-well plates (1 × 10^5^ cells per well) were co-transfected with 1 μg of plasmid expressing IL-1β and 1 μg of plasmid expressing caspase-1/caspase-8/caspase-3 using Lipofectamine™ 3000 Transfection Reagent (Invitrogen, USA) according to the manufacturer’s instructions. Transfected cells were treated with Z-IETD-FMK (IETD, 50 μM) or left untreated. Twenty-four hours post transfection, cells and supernatants were collected for further analysis.

### TUNEL assay

Apoptosis in SARS-CoV-2-infected Calu-3 cells was detected by a TUNEL Assay kit purchased from Thermo Fisher (C10617) according to the manufacturer’s protocols. Briefly, fixed cells were permeabilized with 0.25% Triton™X-100 for 20 min at room temperature. Then the TdT reaction mixture containing TdT and EdUTP was added to label the fragmented DNA in cells. Cells were washed and incubated with Click-iT™ Plus TUNEL reaction cocktail, followed by incubation with DAPI (Beyotime, China) and analyzed using confocal microscope (Andor Dragonfly 202).

### Immunofluorescence assay

Calu-3 cells were mock-treated or infected with SARS-CoV-2 (MOI = 0.1) for 36 h. Cells were fixed and permeabilized, and then blocked with phosphate-buffered saline containing 3% bovine serum albumin. The cells were incubated with rabbit anti-pMLKL (S358, Abcam) and mouse-anti-NP (SR24) for 1 h at room temperature, followed by incubation with Fluor 488 goat anti-mouse IgG and Fluor 561 goat anti-rabbit IgG for 1 h. Cells were washed and incubated with DAPI (Beyotime, China), and then analyzed using confocal microscope (Andor Dragonfly 202).

### Histology and immunohistochemistry

Lung samples obtained from infected and mock-infected mice were fixed with 4% paraformaldehyde, embedded in paraffin and cut into sections of 3.5 μm, and further used for TUNEL assay or processed to IHC. TUNEL assay was performed using an In Situ Cell Death Detection Kit (Roche) according to the manufacturer’s protocols. Briefly, the sections were deparaffinized, rehydrated, and permeabilized with proteinase K. The sections were then incubated with a mixture of TdT and dUTP (2 : 29), followed by incubation with DAPI (Beyotime, China) solution for 10 min. For IHC analysis, the indicated antibodies were used as primary antibodies, then sections were incubated with secondary antibody (Rabbit/Mouse Envision, Dako, Denmark), followed by visualizing with a detection kit (DAB, Dako, Denmark).

The biopsy lung tissue was fixed with 4% paraformaldehyde, paraffin-embedded and cut into sections of 5 μm. For routine histology, sections were stained with HE. Immunostaining was performed using PANO 7-plex IHC kit (Panovue, China) according to the manufacturer’s instructions. TUNEL assay was conducted as described above.

### Statistical analyses

All statistical analyses were performed in GraphPad Prism version 6, as defined in the text and figure legends.

## Supplementary information

Supplemental Material

## Data Availability

The data sets used for the current study are available from the corresponding author upon reasonable request.
