# Supplementary material for: SARS-CoV-2 triggers inflammatory responses and cell death through caspase-8 activation
Source: Signal Transduct Target Ther. 2020 Oct 9;5:235. doi: 10.1038/s41392-020-00334-0 (PMC7545816; doi:10.1038/s41392-020-00334-0)
Supplement: Supplementary file 1 — Supplemental Material [file 41392_2020_334_MOESM1_ESM.docx]

Supplementary Materials for

SARS-CoV-2 triggers inflammatory responses and cell death through caspase-8 activation

Shufen Li, Yulan Zhang, Zhenqiong Guan, Huiling Li, Meidi Ye, Xi Chen, Jun Shen, Yiwu Zhou, Zheng-Li Shi, Peng Zhou, Ke Peng

Correspondence to: pengke@wh.iov.cn; peng.zhou@wh.iov.cn

**This PDF file includes:**

Figures. S1 to S2

Figure. S1


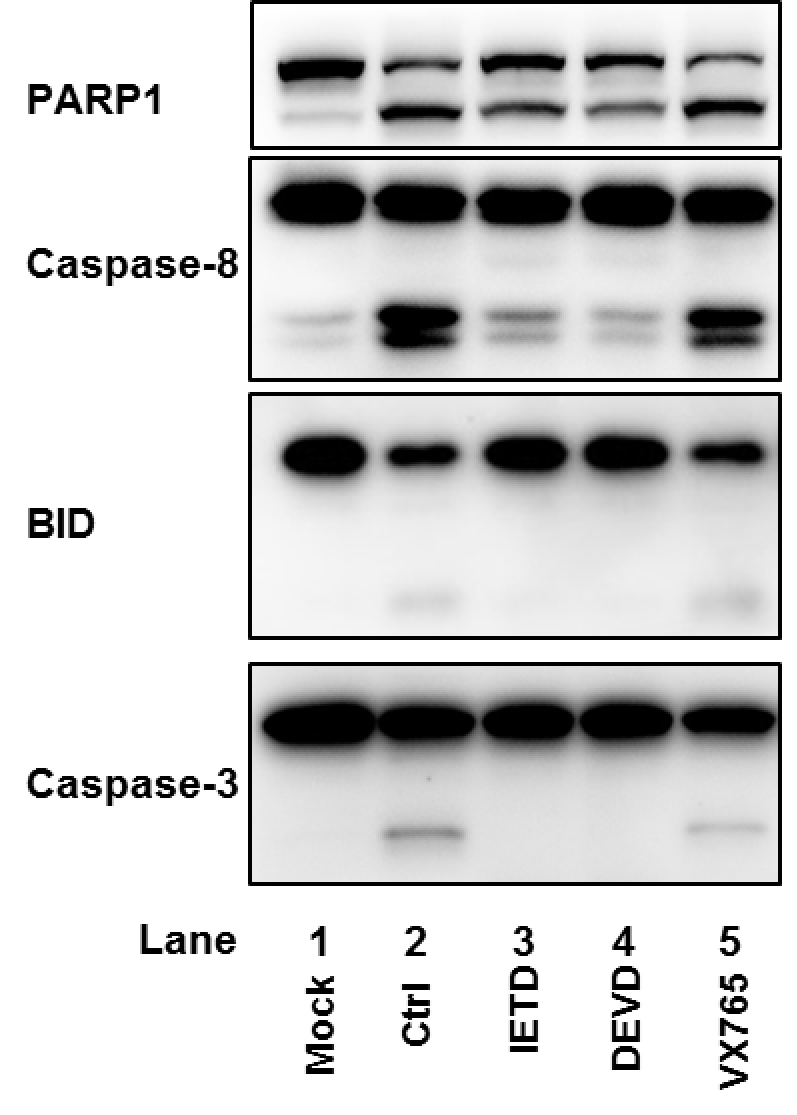


**Fig. S1. Verification of the inhibition effect of Z-IETD-FMK and Z-DEVD-FMK.**Calu-3 cells pre-treated with Z-IETD-FMK (IETD, 50 μM), Z-DEVD-FMK (DEVD, 50 μM) or VX765 (50 μM) were infected by SARS-CoV-2 (MOI=0.03) and subjected to western blot with the indicated antibodies.

Figure S2


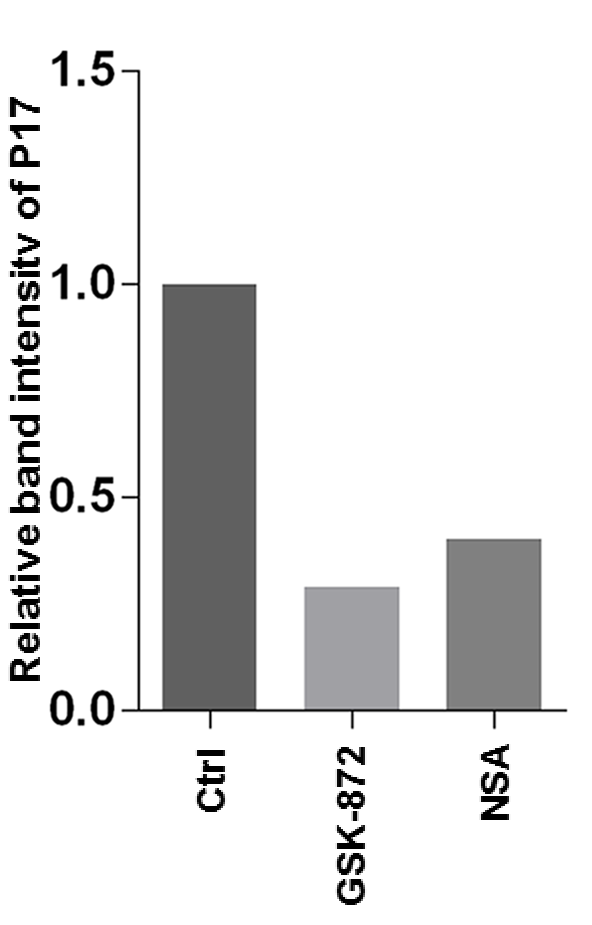


**Fig. S2. Necroptosis pathway mediated IL-1β secretion during SARS-CoV-2 infection.** Calu-3 cells pre-treated with GSK-872 (5 μM) or NSA (2.5 μM) were infected with SARS-CoV-2 (MOI=0.1) for 48 h. P17 levels in the supernatants were determined by western blot. Band intensity of P17 was measured by ImageJ.
